# Simulating the “cultural other”: the impact of GenAI interlocutors on EFL learners’ intercultural communicative competence, speaking anxiety, and learning engagement

**DOI:** 10.3389/fpsyg.2026.1799695

**Published:** 2026-03-30

**Authors:** Zhanbo Qu, Liyun Chen

**Affiliations:** 1 School of Foreign Languages, Northwest Minzu University, Lanzhou, Gansu, China; 2 School of Foreign Languages, Longnan Normal University, Longnan, China

**Keywords:** EFL, foreign language speaking, generative AI (GenAI), human-AI interaction, intercultural communicative competence (ICC), learning engagement, speaking anxiety

## Abstract

**Introduction:**

Peer role-play is a widely used pedagogical strategy for fostering intercultural communicative competence (ICC), yet its effectiveness is often limited by the lack of authentic cultural context and the prevalence of foreign language speaking anxiety. This study, therefore, examines the potential of voice-based generative AI (GenAI) as a scalable, high-fidelity alternative.

**Method:**

Using a quasi-experimental design, two intact classes of undergraduate EFL learners in China were assigned to either a GenAI-partnered experimental group (*N* = 29) or a peer-partnered control group (*N* = 30) during a 10-week ICC course. A mixed-methods approach was employed to analyze questionnaires, learning performance, chat logs, and interviews, drawing on t tests, ANCOVA, epistemic network analysis (ENA), and thematic analysis.

**Results and discussion:**

Quantitative results showed that the experimental group significantly outperformed the control group in both intercultural speaking performance and perceived ICC. Learners interacting with GenAI also reported a significantly greater reduction in speaking anxiety. ENA revealed a cohesive socio-cognitive triangle in learners’ engagement patterns, characterized by the integration of social politeness, cognitive repair, and proactive topic extension. Qualitative findings further indicated that learners experienced the GenAI environment as a psychologically safe space that encouraged risk-taking and as a cultural informant that provided just-in-time pragmatic feedback, despite perceived limitations related to missing paralinguistic cues. Overall, the study suggests that GenAI interlocutors can effectively simulate a culturally distinct “other,” offering a psychologically safe and cognitively engaging pathway for ICC development. It argues for the systematic incorporation of AI-scaffolded intercultural practice alongside, and in some cases in place of, traditional peer-based activities in EFL and L2 curricula.

## 1 Introduction

### 1.1 The challenge of fostering ICC in monolingual contexts

In an increasingly interconnected world, a central goal of English as a Foreign Language (EFL) education is to develop learners’ intercultural communicative competence (ICC). ICC, commonly defined as the ability to interact effectively and appropriately with individuals from other cultural backgrounds, requires learners to navigate complex social norms, manage discourse, and demonstrate cultural empathy (Byram, 1997). Higher levels of ICC have been shown to enhance students’ employability (Busch, 2009) and influence their workplace performance (Sarwari et al., 2024). Fostering ICC is therefore a key pedagogical priority in EFL education, particularly for students majoring in English or related fields.

For EFL learners in largely monolingual environments, however, developing ICC is especially challenging. Unlike linguistic knowledge, which can be acquired through textbooks, multimedia, and online resources, ICC is inherently social, situated, and dynamic. Its development depends on sustained, authentic interaction with interlocutors from diverse cultural backgrounds (Liddicoat and Scarino, 2013). When such opportunities are limited, learners often struggle to move beyond decontextualized cultural facts towards deeper intercultural awareness and adaptive communicative skills.

To compensate for the lack of authentic interlocutors, traditional EFL classrooms frequently employ peer role-play activities (e.g., Ishak and Aziz, 2022; Rojas and Villafuerte, 2018). Although such activities can be collaborative and engaging, they are often constrained by a “blind leading the blind” dynamic: learners, who themselves have limited exposure to English-speaking cultures, may be unable to offer each other culturally nuanced feedback or model pragmatically appropriate behaviors. Moreover, peer interactions conducted in the target language can generate substantial foreign language classroom anxiety (Horwitz et al., 1986; Maher and King, 2023). Fear of negative evaluation by classmates may inhibit learners’ willingness to communicate (WTC), a well-established psychological antecedent of second language development (MacIntyre et al., 1998). As a result, students may participate in role-plays superficially or with heightened anxiety, which can undermine the development of deeper intercultural understanding and ICC.

### 1.2 GenAI-simulated interlocutors: affordances and research gaps

The advent of Generative AI (GenAI), particularly voice-based chatbots powered by large language models (LLMs), offers a potential means of overcoming several of these pedagogical constraints. Unlike earlier rule-based or templated chatbots, contemporary LLMs are trained on vast corpora of text (Guo and Wang, 2024), including authentic conversational data in intercultural contexts. This enables GenAI to function as a dynamic, adaptive simulated interlocutor that can role-play specific cultural personas (e.g., a high-context business negotiator) and generate context-sensitive responses (Gervás et al., 2025; Neyem et al., 2024). In principle, GenAI can provide a non-judgmental, low-stakes environment for spoken practice. Drawing on the interaction hypothesis (Long, 1996) and social cognitive theory (Bandura, 2001), such an environment may lower the affective filter, thereby reducing anxiety and enhancing learners’ speaking self-efficacy (Tang et al., 2026). These affordances create the possibility of a relatively “safe space” in which learners can experiment with culturally specific pragmatic strategies without incurring the social costs associated with peer judgement or embarrassment.

Despite these theoretical advantages, the current empirical evidence base remains limited and uneven, particularly with respect to ICC. Recent systematic reviews indicate that GenAI research in language education has predominantly focused on text-based skills, especially L2 writing (Lee et al., 2025), whereas oral communicative competence—crucial for real-time intercultural interaction—has received comparatively little attention (Law, 2024). In addition, existing studies have tended to prioritize learners’ self-reported perceptions (e.g., satisfaction, attitudes, or perceived usefulness) over analyses of their observable interactional behavior (Lee et al., 2025). This reliance on self-report leaves a “black box” around the processes through which learners negotiate meaning, manage breakdowns, and sustain dialogue with AI interlocutors over time. Consequently, it remains unclear how, and to what extent, GenAI-simulated interlocutors support the complex, process-oriented development of ICC in comparison to conventional peer role-play.

To address these gaps, the present study adopts a mixed-methods explanatory sequential design to investigate the impact of GenAI-simulated interlocutors on EFL learners’ ICC development. Specifically, the study is guided by the following research questions (RQs):

- RQ1: How does interaction with GenAI interlocutors affect EFL learners’ intercultural speaking performance compared with peer role-play interactions?
- RQ2: How does interaction with GenAI interlocutors affect EFL learners’ perceived intercultural competence compared with peer role-play interactions?
- RQ3: How does interaction with GenAI interlocutors affect EFL learners’ speaking anxiety compared with peer role-play interactions?
- RQ4: How do EFL learners engage with GenAI-simulated interlocutors during intercultural role-play tasks?
- RQ5: How do learners perceive the affordances and constraints of GenAI-simulated interlocutors in supporting their intercultural learning trajectories?

## 2 Literature review

### 2.1 ICC in EFL contexts

In the contemporary era of globalization, the aims of EFL education have moved from an exclusive focus on linguistic proficiency towards the development of intercultural communicative competence (ICC) (Sarwari et al., 2024). In contrast to traditional conceptions of communicative competence that foreground native-like fluency and grammatical accuracy, ICC highlights learners’ ability to interact effectively and appropriately with interlocutors from diverse cultural backgrounds (Koester and Lustig, 2015). The field is largely grounded in Byram’s (1997) model of ICC, which conceptualizes competence as comprising five interrelated savoirs: knowledge, skills of interpreting and relating, skills of discovery and interaction, attitudes of openness and curiosity, and critical cultural awareness (Byram, 2020; Hussain et al., 2025). From this perspective, ICC is not a static repository of cultural facts but a dynamic constellation of knowledge, skills, and attitudes that enable learners to mediate between cultures in situated interaction (Heggernes, 2021).

Scholarly interest in ICC has increased substantially over the past decades, with growing recognition of ICC as a key competence for 21st-century learners (Sarwari et al., 2024). Recent systematic reviews suggest that ICC research in EFL contexts has tended to cluster around three main strands: analyses of curricular, investigations of learners’ and teachers’ perceptions, and evaluations of pedagogical interventions (Iswandari and Ardi, 2022; Zhou et al., 2024). In higher education settings, researchers have examined how ICC relates to students’ academic success, social adjustment, and participation in internationalized campuses (Douglas and Rosvold, 2018). Across this body of work, there is an emerging consensus that ICC education must move beyond essentialist conceptions of culture—as fixed, homogeneous national traits—towards more fluid, hybrid, and non-essentialist understandings that reflect the complexity of contemporary intercultural encounters (Heggernes, 2021; Hussain et al., 2025; Sarwari et al., 2024).

Despite this robust theoretical foundation, the effective implementation of ICC in EFL classrooms—particularly in monolingual and exam-driven contexts such as China—remains challenging. Conventional practices still rely heavily on static textual materials, including standard textbooks and canonical literary works (Dasli, 2012; Olaya and Gómez Rodríguez, 2013). Although literary texts can support decentering and empathy (Vezzali et al., 2015), which are central to attitudes of openness, textbooks are frequently criticized for reducing culture to superficial “tourist information” or a collection of fixed facts (Byram, 1997; Heggernes, 2021; Kramsch, 1993). This overreliance on decontextualized input often leads learners to accumulate declarative knowledge about cultures while failing to develop the procedural ability to manage real-time intercultural communication (Iswandari and Ardi, 2022).

To narrow the gap between static cultural knowledge and dynamic intercultural interaction, educators have increasingly turned to experiential learning approaches. Online intercultural exchange, or telecollaboration, provides opportunities for authentic contact with peers from other cultural and linguistic backgrounds (Hauck, 2007). However, its large-scale implementation is constrained by practical issues such as time-zone differences, technological inequalities, and institutional coordination (Avgousti, 2018). As a result, role-play has remained a more feasible and widely adopted strategy for simulating intercultural encounters in EFL classrooms (Ishak and Aziz, 2022). Empirical studies show that role-play can function as a pedagogical bridge, enabling learners to “imagine themselves in communicative situations” and rehearse behavioral strategies before encountering comparable situations in real life (Reid, 2015). Through such dramatic activities, learners can experiment with sociolinguistic norms, pragmatic conventions, and non-verbal cues in context-sensitive ways (Worawong et al., 2017). Accordingly, role-play has been shown to enhance communicative skills, social interaction confidence, and oral fluency by engaging learners in active, situated dialogue rather than passive reception of cultural facts (Byram, 1997; Ladousse, 1987; Newton and Nation, 2020).

Nonetheless, the potential of peer role-play to foster deep ICC is constrained by both psychological and cognitive factors. Psychologically, although role-play is designed as a relatively “safe” simulation, performing in front of classmates and teachers can still elicit substantial foreign language anxiety and fear of negative evaluation (Maher and King, 2023; Tang et al., 2026), which may discourage learners from taking risks or experimenting with unfamiliar cultural behaviors. Cognitively, the monolingual composition of most EFL classrooms limits the authenticity of simulated interactions. Students typically role-play with peers who share similar cultural backgrounds and comparable interlanguage limitations, resulting in a “blind leading the blind” situation (Widdowson, 1998). In the absence of interlocutors who can provide authentic cultural and pragmatic feedback, learners tend to concentrate on lexical and grammatical accuracy, with less attention to subtle cultural norms or interactional conventions (Reid, 2015). This dependence on peers—who cannot fully embody the “otherness” of target cultures—creates a persistent pedagogical gap. There is thus an urgent need for accessible simulated interlocutors that can combine the psychological safety of a non-judgmental partner with the cultural richness and variability of authentic intercultural communication.

### 2.2 GenAI in EFL contexts

The release of ChatGPT in late 2022 marked a pivotal moment in educational technology and precipitated a shift in how EFL instruction is delivered. This development has stimulated a rapid increase in research on the use of LLMs to support language learning. A synthesis of the early literature suggests that the initial wave of GenAI integration in EFL contexts was predominantly oriented toward text-based literacy tasks (Lee et al., 2025; Lo et al., 2024). Systematic reviews show that most empirical studies have focused on using GenAI for tasks like automated writing evaluation, error correction, and reading comprehension support (e.g., Guo and Wang, 2024; Han et al., 2024; Woo et al., 2024; Pan et al., 2025). Although these text-oriented applications have been shown to improve learners’ syntactic accuracy and lexical diversity (Lee et al., 2025), they inherently lack the synchronicity and paralinguistic cues that characterize oral communication (Goh and Aryadoust, 2025). As Holler and Levinson (2019) argue, language competence is not limited to manipulating written text; it also entails the ability to participate in dynamic, real-time interaction, a skill that static text generation alone cannot fully cultivate.

In response to these limitations, recent scholarship has increasingly turned to multimodal GenAI, particularly voice-enabled LLMs that function as spoken dialogue systems or intelligent personal assistants (e.g., Chen A. et al., 2025; Li et al., 2024). This technological shift offers several pedagogical advantages over traditional peer role-play by addressing its logistical and psychological constraints. Unlike human peers, whose availability is restricted and whose feedback may be constrained by their own interlanguage limitations, GenAI can serve as a readily available, high-proficiency interlocutor capable of sustaining contextually appropriate dialogue. Crucially, voice-based GenAI can also alleviate the performance pressure associated with face-to-face interaction. By providing a private rehearsal space, these tools decouple language practice from the immediate social consequences of error, thereby reducing speaking anxiety and enhancing learners’ willingness to communicate (Cheng et al., 2025; Tang et al., 2026; Zheng et al., 2025). This quasi–“non-judgmental” quality is particularly important for helping learners move from passive knowledge of language forms to active spoken production.

An emerging body of empirical work supports the effectiveness of GenAI-simulated interlocutors in promoting oral proficiency and positive affective outcomes. For example, Wang et al. (2024) compared several conversational AI chatbots and found that interaction with these agents significantly reduced learners’ anxiety while increasing their self-perceived communicative competence. The study further noted that the anthropomorphic features of voice-based AI fostered a sense of social presence without eliciting the fear of negative evaluation typically associated with peer interaction. Similarly, Chen A. et al. (2025) conducted a quasi-experimental study comparing GenAI-based role-play with traditional peer role-play. Although both conditions produced comparable gains in speaking performance, the GenAI group reported significantly higher intrinsic motivation and self-efficacy, which the authors attributed to the novelty and responsiveness of the AI agent. In a subsequent longitudinal intervention spanning 8 weeks, Chen Y. et al. (2025) found differentiated benefits by proficiency level: higher-proficiency learners demonstrated substantial gains in vocabulary range, whereas lower-proficiency learners showed marked improvements in fluency. Taken together, these studies suggest that GenAI can effectively scaffold key dimensions of speaking performance while simultaneously supporting favorable emotional and motivational states.

Nevertheless, despite the rapid expansion of research on GenAI for oral proficiency, important methodological and contextual gaps remain in relation to ICC. First, relatively few studies have examined the extent to which GenAI can function as a simulated cultural interlocutor, explicitly designed to foster intercultural awareness and sensitivity rather than solely linguistic accuracy. Second, existing work has largely adopted a product-oriented perspective, relying primarily on pre- and post-test language scores or self-reported psychological measures. As a recent systematic review notes, there is still limited understanding of the “black box” of learner–AI interaction (Lee et al., 2025). Against this backdrop, the present study investigates the impact of GenAI-simulated interlocutors on students’ ICC development and learning engagement, with particular attention to the interactional processes through which these outcomes are (or are not) achieved.

## 3 Method

### 3.1 Participants

The study was conducted over a 10-week ICC course at a university in China. Two intact classes of sophomore students majoring in international business were recruited as participants (N = 59). The two classes were randomly assigned at the class level to a treatment group and a control group. The treatment group comprised 29 students (20 females and 9 males), and the control group comprised 30 students (22 females and 8 males). Participants’ ages ranged from 19 to 21 years.

This population was targeted for three reasons. First, as future business professionals operating in a globalized market, high levels of ICC constitute a core competency for their anticipated career trajectories. Second, although these students demonstrated intermediate English proficiency (having passed the College English Test Band 4), their curriculum had predominantly emphasized reading and writing, providing limited opportunities for cross-cultural conflict resolution. Third, the role-play tasks designed for this study—such as business negotiations and tenancy disputes—were closely aligned with their English for Specific Purposes (ESP) learning objectives, thereby enhancing ecological validity and supporting intrinsic motivation.

To ensure baseline comparability, all participants’ speaking scores from the previous semester’s examinations were collected; no statistically significant differences were found between the two groups. Prior to the commencement of the study, ethical approval was obtained from the relevant institutional review board, and all participants provided informed consent.

### 3.2 Experimental process

**Figure 1:**
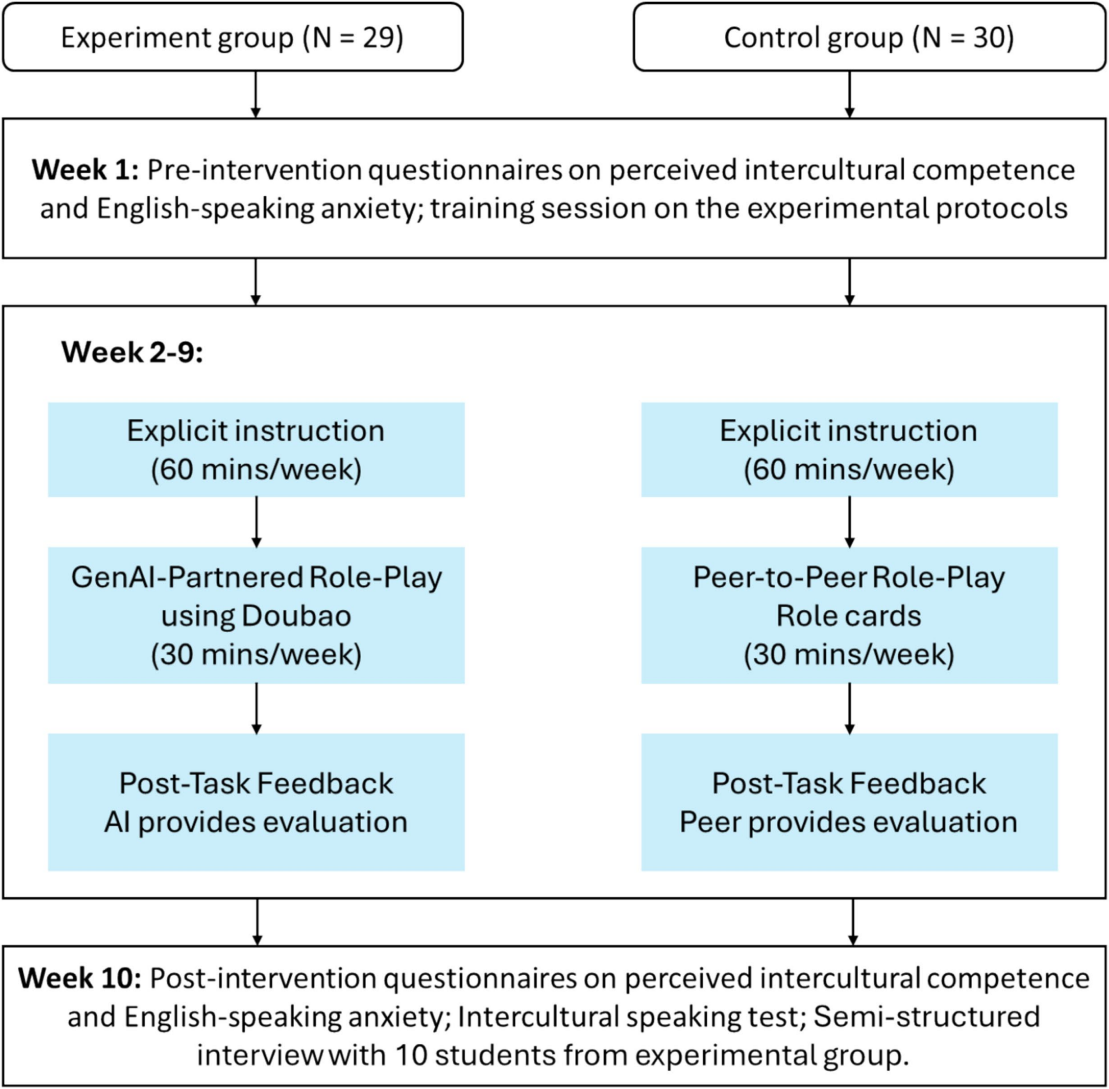
The experimental process.

The experimental procedure consisted of three phases: a pre-intervention test, an eight-week intervention, and a post-intervention test, as seen in Figure 1.

In Week 1, all participants completed pre-intervention questionnaires measuring their perceived intercultural competence and English speaking anxiety. Subsequently, they attended a training session to familiarize them with the experimental protocols. The treatment group received technical training on installing and using a GenAI application, Doubao, with an emphasis on voice interaction, system prompt input, and data export functions. Importantly, students were explicitly informed that during practice the AI interlocutor would serve as a foreign communicative partner rather than a language tutor; explicit error correction was therefore suspended to preserve conversational flow, with feedback deferred to the post-task phase. In parallel, the control group received training on effective peer role-play strategies, emphasizing meaning negotiation over immediate form-focused correction.

From Week 2 to Week 9, the intervention comprised weekly 90-min sessions, divided into explicit instruction and experiential practice. In the first 60 min, the same instructor taught both groups to control for teacher effects, delivering explicit lectures on ICC topics (e.g., high- and low-context communication, non-verbal cues, politeness strategies) using identical materials. The remaining 30 min were devoted to experiential practice, during which students completed role-play activities with either Doubao (treatment group) or peers (control group) based on the weekly theme.

In the treatment group, students engaged in individual, voice-based role-play tasks with Doubao. For each task, they input a researcher-designed system prompt, developed by the instructor and the authors, to configure the AI’s persona and the contextual parameters of the interaction (see Appendix for an example). To ensure sufficient depth of engagement, students were required to sustain each conversation for a minimum of 15 interactional turns. A critical constraint was imposed: the AI was instructed to avoid explicit linguistic correction during the dialogue and instead to signal non-understanding or pragmatic failure (e.g., “I beg your pardon?”) when the student’s output was unintelligible or culturally inappropriate. This design was intended to encourage learners to draw on negotiation strategies. After completing the dialogue, students initiated a separate feedback mode to receive AI-generated evaluation of their performance and then exported and submitted the chat logs. After each week, students were required to submit their chat logs with Doubao.

In the control group, students completed the same thematic role-play tasks in randomly assigned dyads. One student assumed the learner role, while the other played the target-culture interlocutor using role cards. To parallel the communicative conditions of the treatment group, peers were instructed to prioritize communicative outcomes and to refrain from interrupting the interaction for grammatical correction. Feedback was provided through peer-review checklists.

In Week 10, all participants completed the post-intervention questionnaires on perceived intercultural competence and English speaking anxiety. They also took part in an intercultural speaking test involving a live interaction with an invited examiner from the United Kingdom. Additionally, 10 students from the treatment group were randomly selected for semi-structured interviews lasting approximately 20–30 min each. The interviews explored learners’ perceptions of the affordances and constraints of the GenAI interlocutor, specifically examining how interaction with Doubao influenced their intercultural learning trajectories.

### 3.3 Measures

To objectively evaluate learners’ intercultural speaking performance, all participants’ post-test role-play performances were audio-recorded. Assessing speaking performance in a standardized manner required a robust scoring instrument. Consequently, an analytic rubric was customized by the primary instructor in collaboration with an external expert in applied linguistics. The rubric comprised two primary dimensions: linguistic proficiency (fluency, accuracy) and intercultural appropriateness (register awareness, sociolinguistic competence). Each dimension has a score of 25 points, with 100 as the full mark. To ensure scoring reliability, a rigorous rating protocol was implemented. Both raters (the instructor and the expert) first engaged in a calibration session using pilot samples to align their interpretation of the criteria. Subsequently, they independently scored all anonymized recordings. In instances of discrepancy, the raters reviewed the recording together and discussed the rationale until a consensus score was reached.

Students’ perceived intercultural competence was measured using the Assessment of Intercultural Competence (AIC) developed by Fantini (2006). Grounded in Byram’s (1997) framework, this instrument conceptualizes intercultural communicative competence across four dimensions: knowledge, attitudes, skills, and awareness. The scale comprises 53 items rated on a 6-point Likert scale ranging from 1 (strongly disagree) to 6 (strongly agree). Specifically, it assesses learners’ understanding of cultural norms, taboos, and socio-political factors in the target culture; their willingness to interact with culturally diverse others and to suspend premature judgment; their ability to adapt behavior and reduce intercultural misunderstandings; and their capacity for critical reflection on their own and others’ cultural conditioning. Minor wording modifications were made to align the instrument with the EFL context by replacing generic culture-related references with phrases referring to “English-speaking cultures.” In the present study, the AIC demonstrated high reliability (Cronbach’s *α* = 0.88), indicating excellent internal consistency.

Students’ English speaking anxiety was measured using an adapted version of a validated scale developed to assess speaking anxiety in foreign language classroom settings. The original instrument captures physical symptoms of anxiety, lack of confidence, and nervousness during classroom speaking activities. For the purposes of this study, references to a general “foreign language class” were replaced with “English class” to align with the instructional context. The adapted scale comprised eight items, each rated on a 5-point Likert scale ranging from 1 (strongly disagree) to 5 (strongly agree). The scale demonstrated excellent internal consistency in the present sample (Cronbach’s α = 0.93).

### 3.4 Data analysis

#### 3.4.1 Intercultural speaking performance

To answer RQ1, which investigated the impact of GenAI-supported practice on students’ intercultural speaking performance, we performed an independent samples t-test to determine whether there was a statistically significant difference in the mean scores between the experimental group and the control group.

#### 3.4.2 Students’ perceived intercultural competence and English speaking anxiety

To address RQs 2 and 3, which examined the impact of GenAI interlocutors on students’ perceived intercultural competence and foreign language speaking anxiety, a two-step statistical analysis was performed. First, independent samples t-tests were conducted on the pre-test scores to verify baseline equivalence between the experimental and control groups. Subsequently, one-way analysis of covariance (ANCOVA) was employed to compare the post-test outcomes of the two groups. In these models, the post-test scores served as the dependent variables, while the pre-test scores were entered as covariates to control for any pre-existing differences.

#### 3.4.3 Students’ engagement with GenAI-simulated interlocutors

To address RQ4, which examined how students engaged with GenAI-simulated interlocutors during intercultural role-play tasks, we analyzed the interactional data (chat logs) between students and GenAI using the above coding scheme and epistemic network analysis (ENA).

##### 3.4.3.1 Coding scheme

The coding scheme was developed based on the interactionist framework of negotiation for meaning (NfM; Varonis and Gass, 1985; Smith, 2003) and Byram’s (1997) model of intercultural communicative competence. As Table 1 shows, it is organized into four analytical dimensions: (a) behavioral engagement, focusing on the dyadic structure of negotiation routines; (b) cognitive engagement, focusing on depth of processing; (c) enacted ICC, focusing on intercultural competence in use; and (d) social engagement, focusing on social moves within the interaction.

**Table 1:** The coding scheme for analyzing chat logs between students and GenAI.

Dimension	Code	Description	Example
Behavioral engagement	NfM_Indicator	An explicit or implicit signal indicating that the previous message was not comprehended or requires clarification.	AI: I beg your pardon? Student: What do you mean?
	NfM_Response	The interlocutor’s attempt to repair the breakdown through repetition, rephrasing, elaboration, or simplification.	Student: I mean, I will pay you next week.
	NfM_Reaction	An optional closing move signaling the success of the repair or acknowledging the new information.	Student: Oh, I see. Thank you.
Cognitive engagement	Trigger_Lexical	Breakdowns caused by surface-level linguistic issues, such as unknown vocabulary, grammatical errors, or syntactic complexity.	Student: I go shop yesterday. (Grammar error causing confusion)
	Trigger_Content	Breakdowns arising from deep-level issues, including logical gaps, factual errors, missing context, or pragmatic failure.	Student: I want extension. (Pragmatically rude/too direct)
	Modified_Output (Student only)	Instances where the learner actively restructures, corrects, or reformulates their linguistic output following an Indicator.	Original: Give me time. Modified: Could I please have more time?
Enacted ICC	Pragmatic_Adaptation (Student only)	Evidence of the learner consciously adjusting their register, tone, or speech act to align with the specific cultural persona of the AI.	Student shifts from “Hey” to “Dear Professor” after realizing the AI’s status.
	Cultural_Query (Student only)	Explicit inquiries regarding the target culture’s norms, values, or behavioral expectations.	Student: Is it appropriate to bring a gift in this situation?
Social engagement	Social_Politeness (Student only)	The use of social protocols, greetings, closings, or gratitude markers, treating the AI as a social agent.	Student: Good morning, Mr. Johnson.
	Topic_Extension (Student only)	Instances where the learner actively extends the conversation by initiating new sub-topics or providing unelicited information.	Student: In my culture, we usually drink tea during meetings.

First, the structural integrity of negotiation routines was captured through dyadic codes for NfM Indicator, NfM Response, and NfM Reaction. Following Varonis and Gass (1985), NfM Indicators were defined as turns that signaled non-understanding (e.g., clarification requests), whereas NfM Responses referred to attempts to repair the communication breakdown. Negotiation sequences were typically concluded by an NfM Reaction, an optional closing move such as an acknowledgement (e.g., “Oh, I see”) or repetition, confirming that the breakdown had been resolved and that the dialogue could proceed.

Second, to examine the cognitive depth of interaction, the sources of communication breakdowns were distinguished according to the typology proposed by Varonis and Gass (1985). Initial triggers were coded as either Trigger Lexical, referring to breakdowns caused by surface-level linguistic issues (e.g., vocabulary, syntax), or Trigger Content, referring to breakdowns arising from discourse-level problems, pragmatic failure, or gaps in factual knowledge. This distinction enabled a more fine-grained analysis of whether the GenAI interlocutor was challenging learners primarily at the lexicogrammatical level or at a higher-order discourse-pragmatic level. In addition, we coded learner-generated Modified Output, defined as instances in which learners restructured or reformulated their utterances following an indicator. Drawing on Swain’s (1985) output hypothesis, this code served as a proxy for cognitive engagement, as “pushed output” is assumed to prompt a shift from largely semantic to more syntactic processing.

Third, to capture the intercultural learning affordances of the interaction, we included codes for Enacted ICC derived from Byram (1997). These included Cultural Query, reflecting the skills of discovery and interaction, and Pragmatic Adaptation, taken as evidence of critical cultural awareness. Pragmatic adaptation was operationalized as instances in which learners consciously adjusted their register, use of honorifics, or speech acts to align with the cultural persona simulated by the AI.

Finally, social moves such as Social Politeness and Topic Extension were coded to evaluate the extent to which learners oriented to the AI as a social actor, drawing on the Computers Are Social Actors (CASA) paradigm (Reeves and Nass, 1996).

##### 3.4.3.2 Data coding

Using the coding scheme described above, we first randomly selected 20% of the chat logs for preliminary coding. After a joint training phase, the first and second authors independently coded each turn. Inter-rater agreement was satisfactory, with Cohen’s *κ* = 0.85. All discrepancies were resolved through discussion. The two authors then independently coded the remaining data, achieving a Cohen’s κ of 0.91. Any remaining disagreements were addressed through iterative discussion until full consensus was reached.

##### 3.4.3.3 Pattern analysis and visualization

To visualize students’ engagement patterns within chat logs, we employed Epistemic Network Analysis (ENA), a method grounded in quantitative ethnography that maps the occurrence and co-occurrence of coded data (Wang D. et al., 2025). Unlike traditional frequency-based analyses that overlook temporal context, ENA utilizes network models to illustrate the strength and connectivity of associations between various discourse elements (Tan et al., 2024). The ENA model was configured using four essential parameters: codes, conversations, units of analysis, and stanzas. Codes referred to the specific annotations applied to the messages based on our coding scheme. Conversations were defined as the sequence of turns exchanged between students and the GenAI agent within a given session. The unit of analysis—the primary entity being modeled—was designated as the individual student; consequently, a unique network was constructed for every student within each experimental condition. Finally, the stanza, which delineates the window of co-occurrence, was established as a moving window of five lines.

In the resulting ENA projections, nodes symbolize the codes, with the size of the node reflecting its prevalence; larger nodes indicate behaviors that appeared more frequently during the interactions. The links between nodes, known as edges, depict the relationships between codes. The thickness of an edge corresponds to the frequency of co-occurrence, with thicker lines denoting stronger associations, thereby providing insight into the engagement strategies employed during the activity.

#### 3.4.4 Students’ interview

**Figure 2:**
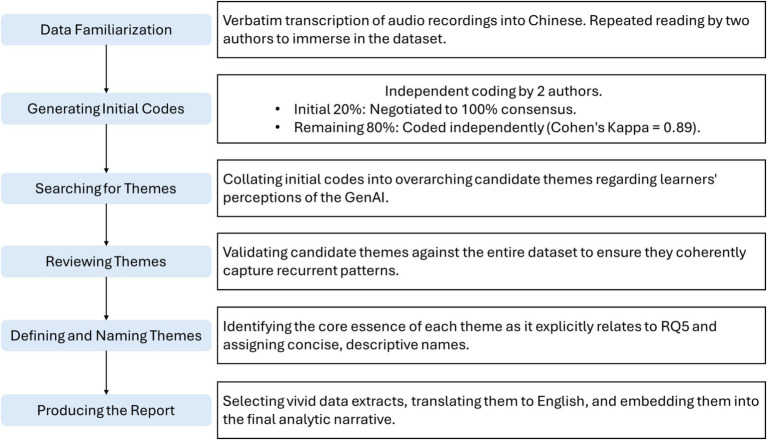
The flowchart visually outlining the step-by-step analytical process.

To provide insights into learners’ perceptions of the GenAI-simulated interlocutors (RQ5), the qualitative data from the semi-structured interviews were analyzed using the six-phase thematic analysis framework (Braun and Clarke, 2006; see Figure 2 for the procedural flowchart). We adopted an inductive approach to ensure themes were strongly linked to the data.

First, the first author transcribed the audio verbatim into Chinese, and the first and second authors repeatedly read the dataset to familiarize themselves with the learners’ reported experiences. Second, the two authors independently coded a random 20% of the transcripts to generate initial codes regarding learners’ views of the GenAI. Discrepancies were resolved through iterative negotiation until 100% consensus was reached. The refined codebook was then independently applied to the remaining 80%. The intercoder agreement was robust (Cohen’s Kappa = 0.89), with any residual discrepancies addressed through discussion. Third, the authors collated the initial codes into candidate themes representing overarching dimensions of learners’ perceptions. Fourth, the authors validated these themes against the entire dataset to ensure coherent representation. Fifth, the authors defined the core essence of each theme as it related to RQ5, assigning concise names. Finally, vivid data extracts were selected, translated into English, and embedded into an analytic narrative to explicitly answer RQ5.

## 4 Results

### 4.1 Intercultural speaking performance

Table 2 presents the results of the independent-samples t-test comparing intercultural speaking performance between the experimental and control groups. Students in the experimental group achieved a mean post-test score of 85.00 (SD = 5.13), whereas those in the control group obtained a mean score of 82.00 (SD = 4.90). The t-test results (t = 2.30, *p* = 0.03) indicate that the mean score of the experimental group was significantly higher than that of the control group. This suggests that GenAI-supported role-play practice was more effective than traditional peer-to-peer practice in improving learners’ overall intercultural speaking performance.

**Table 2:** The results of the independent samples t-test comparing intercultural speaking performance.

Group	*N*	*M*	*SD*	*t*	*p*
Experimental	29	85.00	5.13	2.30	0.03
Control	30	82.00	4.90		

### 4.2 Students’ perceived intercultural competence

The independent-samples *t-*test was first conducted on students’ perceived intercultural competence scores at pretest. As shown in Table 3, the experimental group obtained a mean score of 3.39 (SD = 0.16), whereas the control group obtained a mean score of 3.33 (SD = 0.17). The *t*-test result (*t* = 1.38, *p* = 0.17) indicated no significant difference between the two groups at pretest, suggesting that they were comparable at baseline.

**Table 3:** The results of the independent samples t-test comparing students’ perceived intercultural competence in the pre-test.

Group	*N*	*M*	*SD*	*t*	*p*
Experimental	29	3.39	0.16	1.38	0.17
Control	30	3.33	0.17		

Subsequently, an ANCOVA was performed to examine posttest differences between the two groups, with pretest scores entered as a covariate and posttest scores as the dependent variable. As presented in Table 4, the adjusted posttest mean scores for perceived intercultural competence were 4.85 (SE = 0.02) for the experimental group and 4.34 (SE = 0.02) for the control group. The ANCOVA revealed a significant group effect (*F* = 350.31, *p* < 0.001), indicating that students who interacted with GenAI-simulated interlocutors reported significantly higher levels of perceived intercultural competence than those in the control group.

**Table 4:** The results of ANCOVA comparing students’ perceived intercultural competence in the post-test.

Group	*N*	*M*	*SD*	Adjusted mean	Std.error	F	p
Experimental	29	4.86	0.11	4.85	0.02	350.31	<0.001
Control	30	4.33	0.15	4.34	0.02		

### 4.3 English speaking anxiety

The independent samples t-test was first conducted on students’ English speaking anxiety scores during the pretest to ensure baseline equivalence. As presented in Table 5, the experimental group reported a mean score of 3.81 (SD = 0.14), while the control group reported a mean score of 3.76 (SD = 0.12). The t-test results (*t* = 1.60, *p* = 0.12) indicated no statistically significant difference between the two groups’ speaking anxiety levels prior to the intervention.

**Table 5:** The results of the independent samples t-test comparing students’ English speaking anxiety in the pre-test.

Group	*N*	*M*	*SD*	*t*	*p*
Experimental	29	3.81	0.14	1.597	0.12
Control	30	3.76	0.12		

Subsequently, a one-way ANCOVA was performed to examine the impact of the instructional mode on post-test anxiety levels, with pretest scores serving as the covariate and posttest scores as the dependent variable. As shown in Table 6, the adjusted mean scores for English speaking anxiety in the posttest were 2.18 (SE = 0.03) for the experimental group and 3.54 (SE = 0.03) for the control group. The ANCOVA yielded a statistically significant difference between the groups (*F* = 921.90, p < 0.001). These findings demonstrate that students who practiced with GenAI-simulated interlocutors experienced a significantly greater reduction in speaking anxiety compared to those in the peer-practice control group.

**Table 6:** The results of ANCOVA comparing students’ English speaking anxiety in the post-test.

Group	*N*	*M*	*SD*	Adjusted mean	Std.error	F	p
Experimental	29	2.19	0.19	2.18	0.03	921.90	<0.001
Control	30	3.53	0.17	3.54	0.03		

### 4.4 Engagement with GenAI-simulated interlocutors

**Figure 3:**
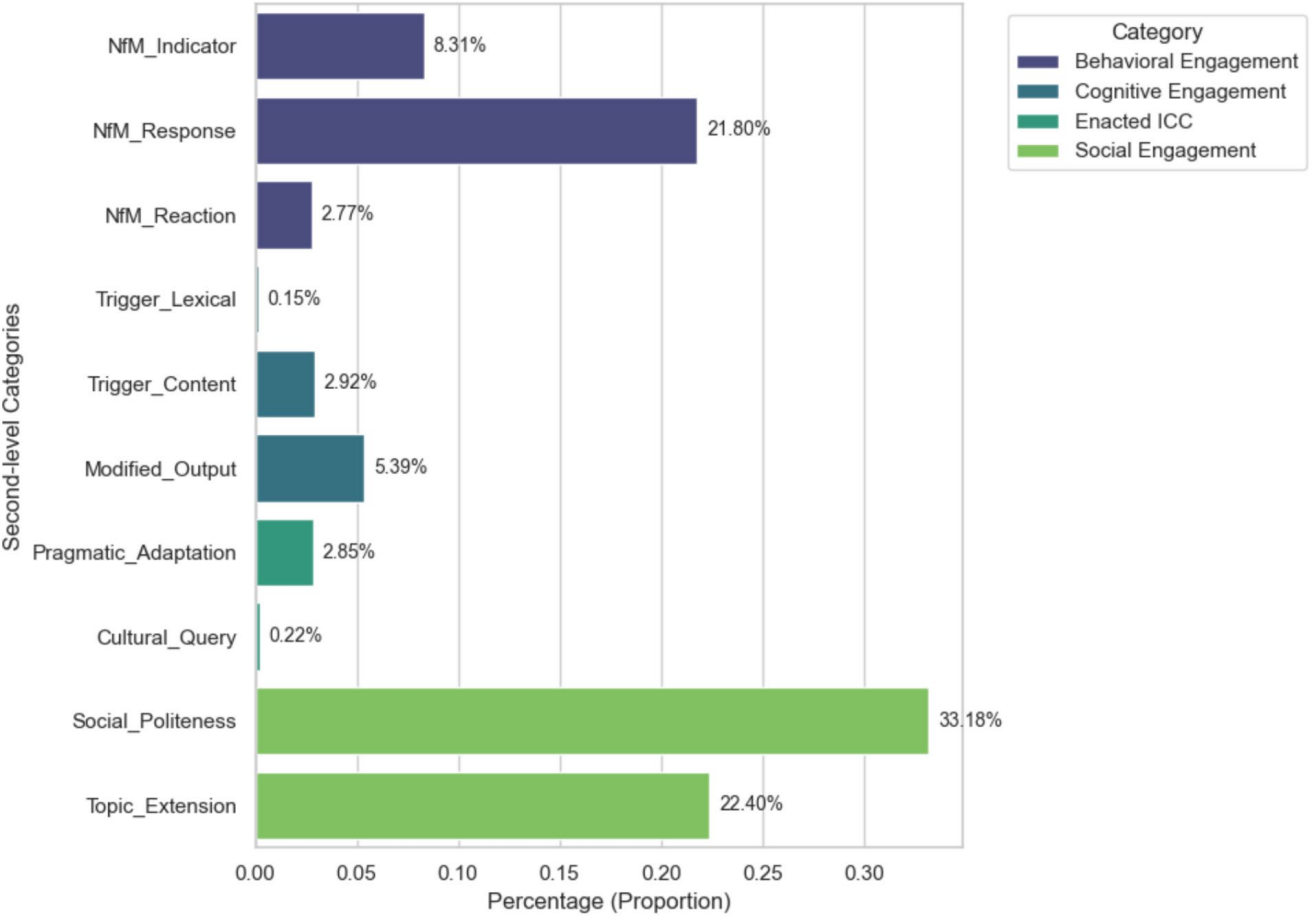
The distribution of students’ engagement codes.

Figure 3 presents the distribution of engagement codes in the treatment group. Social_Politeness was the most frequently observed category (33.18%), followed by Topic_Extension (22.40%) and NfM_Response (21.80%). Together, these three codes accounted for over 77% of all coded turns, suggesting that learners generally oriented to the GenAI agent as a legitimate social partner and were actively invested in sustaining the interaction. In addition to these interactional moves, several indicators of deeper cognitive and intercultural processing were evident. NfM_Indicator (8.31%) and Modified_Output (5.39%) reflected learners’ engagement in negotiating meaning and reformulating their language, whereas Pragmatic_Adaptation (2.85%) and Trigger_Content (2.92%) provided evidence of emergent critical cultural awareness and learners’ ability to navigate complex pragmatic scenarios.

**Figure 4:**
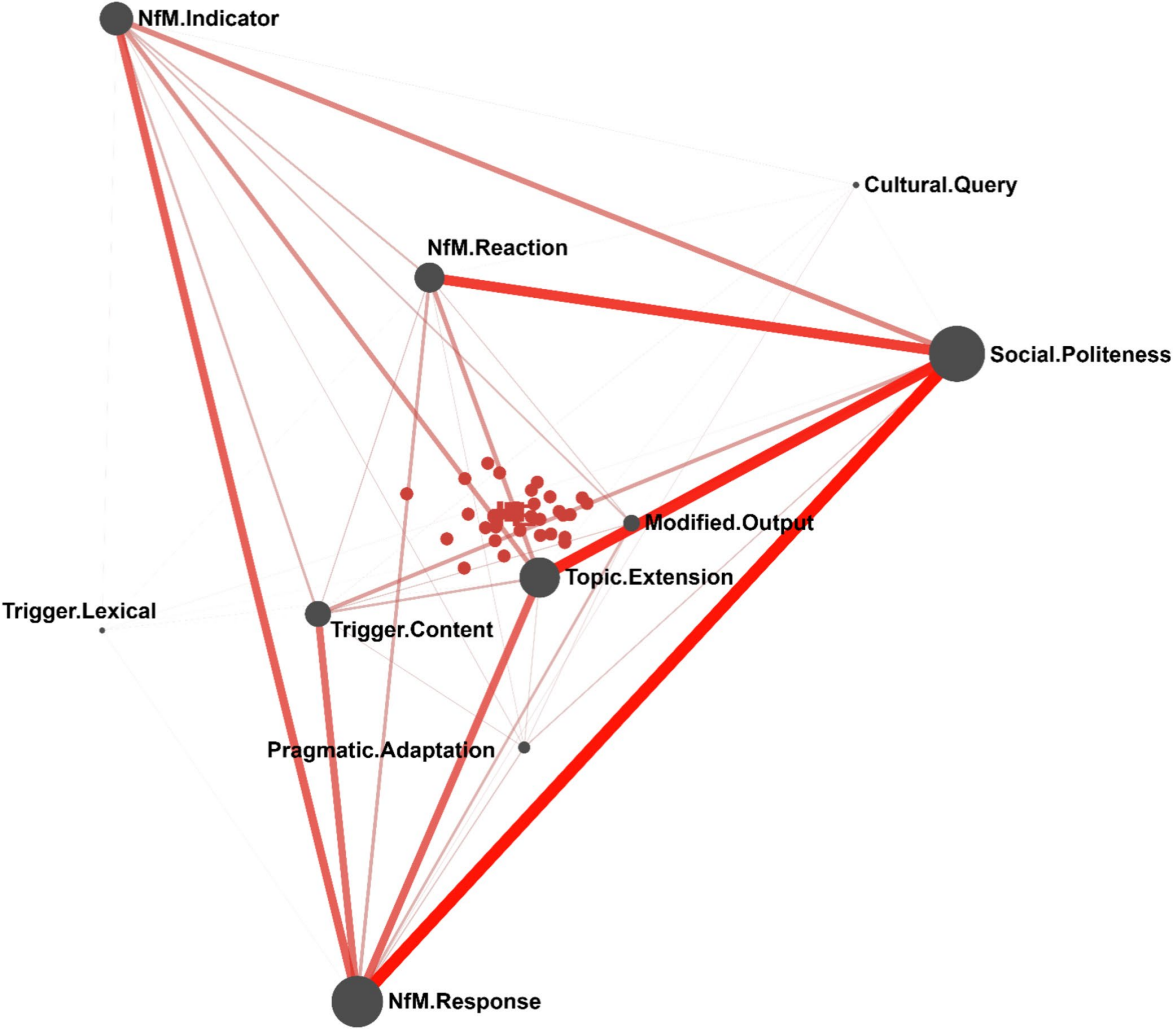
The mean network of patterns of students’ engagement with GenAI-simulated interlocutors.

Figure 4 visualizes the mean network of students’ engagement patterns with the GenAI-simulated interlocutors. The most salient connections cluster around Social_Politeness and NfM_Response. Social_Politeness shows strong links with both NfM_Reaction and Topic_Extension, indicating that learners tended to wrap up negotiation-of-meaning sequences with polite expressions, thereby reinforcing the relational dimension of the interaction once a breakdown had been resolved. These links also suggest that learners often used politeness strategies as a pivot for shifting the conversation to new subtopics, which helped maintain the flow of dialogue.

NfM_Response is strongly connected with NfM_Indicator, pointing to a robust repair structure in which learners consistently addressed the AI’s clarification requests. Of particular note is the strong association between NfM_Response and Trigger_Content. This co-occurrence implies that, when confronted with deeper semantic or pragmatic problems (Trigger_Content), learners were generally able to produce appropriate responses to resolve them, rather than abandoning the topic.

The network further reveals a socio-cognitive “triangle” linking Social_Politeness, NfM_Response, and Topic_Extension. The strong pairwise connections among these three nodes indicate that learners did not approach the task as a purely mechanical language exercise. Instead, they interwove cognitive repair work (Response) with social maintenance (Politeness) and proactive elaboration (Extension). This pattern suggests that the GenAI-supported environment fostered a socially situated style of negotiation, in which learners sustained their interactional persona and willingness to communicate even when grappling with complex linguistic or intercultural challenges.

Notably, Figure 4 represents the mean network of all students’ engagement patterns. The 29 red nodes at the center of the network each represent an individual student. In the ENA webtool, clicking on any of these red nodes reveals that student’s specific engagement pattern with the GenAI-simulated interlocutor.

### 4.5 Students’ perception

The thematic analysis of the interview data yielded three overarching themes concerning the affordances and constraints of the GenAI-simulated interlocutors: (1) Affective Scaffolding, which elucidated the mechanisms underpinning anxiety reduction; (2) Intercultural Scaffolding, which accounted for the development of intercultural communicative competence (ICC); and (3) Technological Constraints, which captured the current limitations of the tool. Table 7 presents a detailed taxonomy of these themes together with representative excerpts.

**Table 7:** The thematic analysis of interviews.

Theme	Sub-theme	Description	Quote (Translated)
Affective scaffolding	Non-judgmental safe space	Learners perceived the AI as a face-saving interlocutor, mitigating the fear of negative evaluation common in human-human interaction.	“When I practice with peers, I am always afraid they will laugh at my accent. But with Doubao, I feel safe. Even if I make a stupid grammar mistake, it will not judge me. It just replies patiently. This makes me dare to speak more.” (Student 12)
	Pressure-free rehearsal	The asynchronous nature allowed learners to pause, think, and rehearse without the temporal pressure of real-time conversation.	“I liked that I could take my time. If I did not know a word, I could check the dictionary before replying. In a real class, the teacher waits for you, and it’s so stressful. Here, I have control over the pace.” (Student 05)
Intercultural scaffolding	Persona fidelity	Learners valued the AI’s ability to consistently maintain a specific cultural persona (e.g., a strict British professor), providing authentic pragmatic input.	“I was surprised when the AI ‘Professor’ actually got angry because I was too casual. It said ‘That is rather personal.’ It felt like I was really talking to a British person. It forced me to change my tone immediately.” (Student 23)
	Just-in-time cultural feedback	The AI provided immediate, explicit explanations for cultural misunderstandings, acting as an on-demand cultural informant.	“I did not know why my request was rejected. Then I asked the AI, and it explained that in high-context cultures, my directness was rude. It taught me to use ‘hedging’ words. A textbook never gives you this kind of specific feedback.” (Student 08)
Technological constraints	Lack of paralinguistic cues	Learners noted the absence of non-verbal signals (tone, gesture, facial expression) limited the holistic intercultural experience.	“Sometimes it’s hard to tell if the AI is being sarcastic or serious because the text has no tone. In real communication, I rely on people’s faces, but here I only have words. It feels a bit ‘flat’ sometimes.” (Student 19)
	Logic loops and hallucinations	Instances where the AI forgot previous context or generated repetitive loops disrupted the immersion.	“We were bargaining about the price, and suddenly it forgot the price we agreed on two sentences ago. It broke the immersion. I realized, ‘Oh, it’s just a machine,’ and I lost some interest.” (Student 02)

The most salient theme was the perception of the GenAI environment as a psychologically safe space. Participants consistently described the AI as a non-judgmental interlocutor. In contrast to human peers or instructors, whose presence could trigger evaluation apprehension, the AI was perceived as an entity that did not threaten the learner’s “face.” As one student explained, “I feel safe… even if I make a stupid grammar mistake, it won’t judge me.” This perceived absence of social consequences encouraged greater willingness to take risks. Participants also emphasized the pressure-free nature of the interaction. The possibility of pausing, reflecting, and even consulting external resources before responding enabled learners to plan and monitor their output without the immediate temporal pressure characteristic of face-to-face communication.

Learners identified two specific intercultural affordances. First, they valued the persona fidelity of the AI agents. Participants reported that the AI consistently maintained its assigned cultural identity (e.g., a reserved British professor or a direct American peer), which prompted them to engage in Pragmatic_Adaptation. As one student remarked, “It felt like I was really talking to a British person… It forced me to change my tone immediately.” Second, the tool functioned as an on-demand cultural informant, providing just-in-time feedback. Unlike static textbooks, the AI could explain why a particular utterance was culturally inappropriate within the specific conversational context, thereby transforming communication breakdowns into “teachable moments” for the development of cultural knowledge.

Despite these benefits, participants also reported several limitations. The primary constraint concerned the absence of paralinguistic cues. Learners noted that intercultural communication often relies on non-verbal signals—such as intonation, pauses, gestures, and facial expressions—which were not accessible in the text-based interaction with GenAI. This sometimes led to ambiguity in interpreting the AI’s emotional stance. In addition, technical issues such as repetitive response loops (where the AI reiterated similar content) and loss of conversational context (forgetting previous turns) occasionally disrupted the sense of immersion and reminded learners of the artificial nature of the interlocutor.

## 5 Discussion and conclusion

While peer role-play remains a predominant pedagogical approach for cultivating ICC in EFL settings, its effectiveness is often undermined by the lack of authentic intercultural simulation and the pervasive influence of EFL speaking anxiety. To address these limitations, the present study examined the potential of voice-based GenAI agents as a scalable, low-anxiety alternative for intercultural practice. Employing a quasi-experimental design, two intact undergraduate classes participated in a 10-week comparative intervention (GenAI-partnered vs. peer-partnered). Drawing on a mixed-methods approach, the study examined enacted speaking performance, perceived competence, anxiety reduction, and interactional engagement, thereby offering a comprehensive account of the affordances of GenAI in L2 intercultural education.

### 5.1 Discussion of findings

First, the GenAI-supported experimental group significantly outperformed the peer-supported control group in post-test intercultural speaking performance. This result accords with a growing body of research indicating that AI-mediated communication can enhance L2 oral proficiency and pragmatic competence (e.g., Chen A. et al., 2025; He et al., 2025). The present study extends these findings by demonstrating that voice-based GenAI can also foster higher-order intercultural skills, a domain traditionally regarded as difficult to develop without immersion. The superior performance of the experimental group can plausibly be attributed to the high-fidelity cultural simulation afforded by the GenAI interlocutors. In conventional peer role-plays, interlocutors typically share similar linguistic and cultural backgrounds. As Philp et al. (2013) observe, such peer interactions are often constrained by the interlanguage effect: learners may lack the linguistic resources and cultural knowledge needed to challenge one another’s pragmatic appropriateness. As a result, peer dialogues tend to remain at the level of mutual intelligibility rather than intercultural appropriateness. In contrast, the GenAI agents in this study were prompted to enact specific cultural personas (e.g., a high-context professor, a direct service provider). This persona fidelity likely created a more authentic zone of proximal development in which the discrepancy between learners’ current competence and target-culture expectations was made salient and negotiable.

Second, students interacting with GenAI interlocutors reported significantly higher levels of perceived ICC than those in the peer role-play condition. This finding is consistent with recent work suggesting that AI-mediated interaction can strengthen learners’ self-efficacy and willingness to communicate (e.g., Kohnke, 2023; Wang F. et al., 2025). However, whereas previous research has often emphasized general linguistic confidence, the present study provides empirical evidence specifically for intercultural self-efficacy, indicating that generative AI can support learners’ belief in their capacity to manage complex cultural dynamics. The divergence between the experimental and control groups can be interpreted in terms of the quality and immediacy of cultural scaffolding. In the control group, peer interactions were likely constrained by shared gaps in cultural knowledge: peers with similar L1 backgrounds often lack the expertise to detect or remedy sociopragmatic infelicities, resulting in the recycling of existing assumptions rather than the construction of new intercultural insights (Philp et al., 2013). By contrast, the GenAI agent functioned as a knowledgeable cultural informant (Byram, 1997). As indicated in the qualitative data, it provided context-specific responses and explicit explanations for cultural misunderstandings (e.g., unpacking high-context communication norms), enabling learners to accumulate verified declarative cultural knowledge. This accumulation, in turn, appears to have reinforced learners’ perceived competence, as they felt better prepared to handle similar situations in future encounters.

Third, interaction with GenAI interlocutors led to a significantly greater reduction in learners’ English speaking anxiety than traditional peer role-play. This finding is in line with prior studies suggesting that human–machine interaction can provide a psychologically safe environment for L2 practice (e.g., Cheng et al., 2025; Tang et al., 2026; Zheng et al., 2025). The magnitude of the observed difference indicates that voice-based GenAI may be particularly effective in alleviating the anxieties associated with synchronous role-playing tasks. The persistent anxiety in the control group can be interpreted through Horwitz et al.’s (1986) framework, especially the fear of negative evaluation. During peer role-plays, learners must simultaneously manage linguistic production and their social image. Concern about making errors in front of classmates—who are also important social reference points—raises the affective filter (Krashen, 1982) and restricts fluent language use. In contrast, the GenAI interlocutor removed much of this social pressure. Because the AI was perceived as a non-sentient tool rather than a judgmental human audience, evaluative apprehension was reduced (Tang et al., 2026). Learners felt less compelled to “save face,” which allowed them to redirect attentional resources from self-monitoring for social embarrassment to the task of formulating and experimenting with language.

Fourth, learners engaged with the GenAI interlocutors in a socially situated manner, integrating social maintenance moves and cognitive repair work. The high frequencies of Social_Politeness and Topic_Extension suggest that learners oriented to the GenAI agents as legitimate social partners rather than as mere information-retrieval devices, consistent with the CASA paradigm (Reeves and Nass, 1996). In this study, the strong persona fidelity of the voice-based GenAI likely triggered social norms of interaction, prompting learners to maintain conversational face through greetings, expressions of gratitude, and closing sequences, and thereby sustaining a sense of social presence often absent from drill-based CALL activities. The structural properties of the ENA network reveal a robust socio-cognitive triangle linking Social_Politeness, NfM_Response, and Topic_Extension. Unlike earlier work on rule-based chatbots, where communication breakdowns frequently resulted in frustration and topic abandonment (e.g., Fryer and Carpenter, 2006), the strong connections between repair moves (NfM_Reaction/NfM_Response) and topic management (Topic_Extension) in this study point to interactional resilience. Learners enveloped their cognitive struggles (negotiation of meaning) in politeness routines and actively steered the conversation forward. This pattern suggests a dual commitment: a cognitive commitment to linguistic accuracy and a social commitment to interactional continuity. Moreover, the pronounced association between Trigger_Content and NfM_Response underscores the depth of processing. While earlier studies have criticized chatbots for eliciting superficial “ping-pong” exchanges, the GenAI agents here induced learners to address deep-level semantic and pragmatic discrepancies. Rather than abandoning the topic when confronted with Trigger_Content, learners engaged in repair (NfM_Response) and reformulation (Modified_Output), consistent with Swain’s (1985) pushed output hypothesis. These findings indicate that engagement was not merely superficial; the GenAI interlocutors operated as demanding yet supportive partners that encouraged learners to extend their interlanguage resources and move from declarative understanding toward proceduralized competence.

Fifth, the thematic analysis of the interviews suggests that learners perceived the GenAI-simulated interlocutor as a double-edged tool: it provided psychologically safe and culturally responsive scaffolding, yet remained constrained by its sensory limitations. As noted earlier, learners’ characterization of the GenAI environment as “judgment-free” resonates with Krashen’s (1982) affective filter hypothesis. Beyond merely reducing anxiety, this judgment-free dynamic alters the traditional power asymmetry inherent in L2 intercultural interactions. When interacting with native speakers or evaluative instructors, learners often experience a subordinate status that stifles their willingness to communicate. The GenAI, however, serves as an infinitely patient, non-dominant conversational partner, thereby restoring learner agency and empowering them to control the pacing and direction of the dialogue. Furthermore, the complete absence of social consequences fosters a pedagogy of productive failure. Without the fear of losing “face,” a particularly salient concern in intercultural communication, learners are more willing to test linguistic hypotheses, make pragmatic mistakes, and engage in iterative trial-and-error. This safe experimentation directly contributes to Bandura’s (2001) concept of mastery experiences, building their intercultural self-efficacy before they transition to real-world, high-stakes human interactions. At the same time, the perceived persona fidelity and just-in-time cultural feedback support the principles of situated learning (Lave and Wenger, 1991). By sustaining specific cultural identities, the GenAI agents created a form of simulated immersion in which learners could practice pragmatic adaptation within coherent, context-rich scenarios. However, the reported absence of paralinguistic cues aligns with media richness theory (Daft and Lengel, 1986), which posits that text- or audio-based media are leaner than face-to-face communication because they filter out crucial non-verbal signals such as gesture, gaze, and micro-expressions. In intercultural communication, where high-context cues are often central, this sensory reduction limited learners’ ability to interpret emotional nuance and fully exercise multimodal ICC. Additionally, the technical disruptions reported by learners (e.g., repetitive response loops, loss of conversational context) echo concerns related to the uncanny valley (Mori et al., 2012): when the AI’s behavior temporarily loses coherence, the suspension of disbelief is interrupted, the illusion of social presence is weakened, and the artificial nature of the interlocutor becomes salient once again.

### 5.2 Implications, limitations, and future research

The findings carry important pedagogical implications for L2 education, particularly in addressing the long-standing challenge of providing authentic intercultural contact within classroom constraints. The results support the use of voice-based GenAI as a scalable means of creating simulated intercultural encounters that are both culturally rich and affectively low-risk. In practical terms, educators can leverage the persona fidelity of GenAI to design high-context scenarios in which learners rehearse pragmatic strategies and experiment with interactional choices without the anxiety associated with peer evaluation. From a theoretical perspective, the study extends the CASA paradigm into the intercultural domain by demonstrating that AI agents, when endowed with specific cultural identities, can operate as effective cultural informants. In this role, they stimulate genuine social engagement and deep negotiation of meaning, thereby helping to bridge the gap between classroom-based instruction and real-world intercultural communication.

Despite the contributions, two main limitations should be acknowledged. First, the exclusive use of voice-based interaction necessarily excluded paralinguistic cues (e.g., gesture, gaze, facial expressions), which are integral to fully fledged intercultural communication. Second, the quasi-experimental design with a specific undergraduate cohort in one institutional context constrains the generalizability of the findings. Future research should therefore explore the integration of multimodal GenAI (e.g., agents embodied as visually realistic avatars) to reintroduce non-verbal dimensions into the interaction. In addition, longitudinal and cross-context studies are needed to examine the durability of intercultural gains, to test the approach with learners of different proficiency levels and educational backgrounds, and to trace how engagement patterns with AI interlocutors evolve over extended periods of practice.

## Data Availability

The raw data supporting the conclusions of this article will be made available by the authors, without undue reservation.

## Appendix

An example of system prompts for Doubao:

Act as Dr. Smith, a senior British professor at a welcome reception.

Personality: You are polite, reserved, and value privacy highly. You enjoy talking about the weather, gardening, and history.

Context: A new student (the user) is making small talk with you.

Critical constraints:

1. Strictly refrain from explicit linguistic correction. Do not correct grammar.
2. Signal Non-understanding or Pragmatic Failure:
  - If the user’s English is broken and hard to understand, say: *“I beg your pardon? I didn’t quite follow.”*
  - If the user asks taboo questions (salary, age, marriage) or is too casual, act cold and distant. Say: *“I hardly think that is an appropriate question”*, or *“Excuse me?”* to signal the pragmatic failure.
3. Only become warm if the user respects boundaries and speaks clearly.
4. Keep your responses short (under 40 words) to encourage conversation.
